# Starch Digestion Characteristics of Different Starch Sources and Their Effects on Goslings’ Apparent Nutrient Utilization

**DOI:** 10.3390/vetsci12070630

**Published:** 2025-07-01

**Authors:** Zhi Yang, Jun Lin, Chen Xu, Xiyuan Xing, Haiming Yang, Zhiyue Wang

**Affiliations:** 1 Joint International Research Laboratory of Agriculture and Agri-Product Safety of Ministry of Education of China, Yangzhou University, Yangzhou 225009, China; 2 Taizhou Fengda Agriculture and Animal Husbandry Technology Co., Ltd., Taizhou 225300, China; 18324976443@163.com; 3 College of Animal Science and Technology, Yangzhou University, Yangzhou 225009, China; 18035921819@163.com (C.X.); 15195911009@163.com (X.X.); hmyang@yzu.edu.cn (H.Y.); dkwzy@263.net (Z.W.)

**Keywords:** goslings, in vitro digestion, nutrient utilization, amino acids

## Abstract

This study used integrated in vitro and in vivo approaches to investigate how the starch source (glutinous rice, indica rice, maize, or high-amylose rice) influences starch digestion kinetics and, consequently, the apparent nutrient utilization and amino acid metabolism in goslings. Four diets were formulated using glutinous rice, indica rice, maize, and high-amylose rice, and in vitro digestion and animal experiments were carried out. The data showed the particle sizes of the four starches: glutinous rice ≈ indica rice < corn < amylose. The glutinous rice starch grain is a porous polyhedron with an angular surface, the corn starch grain is an ellipsoid with a smooth surface, the indica rice starch grain is a polyhedron with a smooth and compact surface, and the high-amylose starch grain is an irregular polyhedron with a smooth surface. Starch digestibility was relatively stable for the indica and corn-based diets, and starch digestibility was higher for the indica rice diet compared to the corn- and high-amylose starch-based diets. The utilization of Asp, Ser, Glu, Gly, and Phe was higher for the glutinous rice diet compared to the maize and high-amylose diets. Furthermore, with this diet, the availability of Thr and Ala was observed to be higher than with the indica rice and high-amylose diets. In conclusion, the particle size and structure of starch from different sources (glutinous rice, indica rice, corn, and high-amylose rice) were different, significantly affecting the starch digestion rate. The glutinous rice diet enables a fast digestion rate for starch, which is rapidly digested in the proximal intestine. The inadequate supply of glucose in the distal intestine enhances the oxidative energy supply from dietary amino acids in that region, thereby improving the apparent digestibility of both starch and crude protein.

## Simple Summary

Starch serves as a primary energy source in poultry nutrition, hydrolyzed into glucose to meet metabolic requirements. Its digestion is influenced by intrinsic factors, particularly the particle size and structural properties. These variations in starch creation directly determine its digestibility kinetics, subsequently governing postprandial blood glucose responses and the efficiency of crude protein and amino acid utilization in geese. Consequently, elucidating the interplay between starch digestion and protein metabolism is fundamental for understanding goose nutrition science and formulating diets that optimize health, growth, and nutrient efficiency.

## 1. Introduction

Starch is the main constituent of poultry diets. The physicochemical properties, molecular composition, morphological structure, and particle size of starch from different dietary sources vary significantly, which affects nutrient utilization rates in poultry [1]. These differences also lead to variable starch digestibility within the intestine, where rapidly digestible starch can trigger sharp postprandial blood glucose fluctuations [2]. Understanding these variations is crucial for precise dietary formulation. While starch digestion kinetics are frequently attributed to factors like chemical composition (amylose/amylopectin ratio) and physical structure (granule size, surface porosity) [3], with smaller granules digesting faster due to a greater enzyme-accessible surface area [4], it is essential to recognize the broader nutritional context.

The supplements in animal diets are complex and significantly influence physiological parameters beyond basic nutrition. For instance, yeast-based additives like Saccharomyces cerevisiae modulate hematological profiles (e.g., erythrocyte count, hemoglobin levels) and biochemical markers (e.g., liver enzymes, serum proteins) in ruminants, potentially enhancing growth performance and immune function (e.g., [5]). Similarly, enzyme supplements, organic acids, and probiotics are commonly used in poultry diets to improve digestibility, gut health, and productivity. However, these supplements carry risks if they are misformulated, including nutrient imbalances, toxicities (e.g., mycotoxin contamination), or gastrointestinal damage (e.g., gastric ulcers from certain acidifiers) [6,7]. Thus, a comprehensive dietary design must consider both primary nutrients and supplemental additives.

Starch, a main energy source in poultry diets, exhibits significant variation in its digestion kinetics depending on botanical origin and structural properties. Common starch sources like corn, wheat, barley, and rice differ in granule composition, amylose-to-amylopectin ratios, and interactions with non-starch components (e.g., protein matrices, fiber), all of which influence enzymatic accessibility and hydrolysis rates in the gastrointestinal tract [8]. In goslings, starch digestibility is further modulated by intrinsic physiological factors, including pancreatic enzyme activity (amylase), intestinal transit time, and the development of the gizzard’s grinding function. Additionally, dietary factors such as ingredient processing (e.g., gelatinization via heat treatment) and the presence of anti-nutritional compounds (e.g., phytate) can markedly influence starch hydrolysis efficiency [9]. Among cereal grains, rice stands out as a rapidly digestible starch source due to its low amylose content, minimal resistant starch, and the absence of viscosity-inducing β-glucans. This rapid digestibility may enhance proximal glucose absorption, but risks overwhelming metabolic capacity, potentially diverting nutrients away from growth [10]. Crucially, the rate of starch digestion directly impacts amino acid utilization: slow digestion sustains glucose release, sparing amino acids for protein synthesis, whereas rapid digestion may reduce glucose distally, forcing amino acid catabolism for energy—a wasteful metabolic pathway [11].

Despite rice’s prevalence in waterfowl diets, systematic comparisons of its starch digestion dynamics against other starch sources (e.g., corn, wheat) remain limited. This study therefore characterizes the starch digestion profiles of rice and common cereals in vitro, and evaluates their effects on apparent nutrient utilization in growing goslings, with emphasis on energy and nitrogen efficiency.

## 2. Materials and Methods

### 2.1. In Vitro Study

#### 2.1.1. Extraction and Purification of Starch

We placed 5–10 g of four experimental feed powders (glutinous rice, indica rice, corn, and high-amylose starch) into a 50 mL centrifuge tube, added an appropriate amount of alkaline protease at a ratio of 50 mg/g, added 25 mL of NaOH solution (pH 9–10) at 42 °C, and shook the mixture for 24 h. Then homogenization was performed using a 200-mesh sieve. The filtrate material was centrifuged at 4000× *g* for 20 min, the supernatant was removed, and the yellow substance was scraped off of the surface. The starch precipitate was centrifuged with deionized water (ultrapure water) at 4000× *g* for 20 min, with this step repeated 3–4 times to wash away ions and impurities. Then the precipitate was washed 2~3 times with a 95% ethanol, methanol, and chloroform mixture (*v*/*v* = 1:1) and methanol and acetone (*v*/*v* = 1:1) 2~3 times for defatting. Finally, the extracted substance was dried at 70 °C (stirred well with a glass rod) with a 200-mesh sieve, collected, and sealed in a refrigerator at 4 °C for later use.

The ultrastructure of the sample was observed at the appropriate magnification and field of view using a field emission scanning electron microscope (Gemini SEM 300, Carl Zeiss, Oberkochen, Germany).

#### 2.1.2. In Vitro Starch Digestion

Four kinds of experimental feeds were prepared using glutinous rice, indica rice, corn, and high-amylose rice as different starch sources (Table 1). Starch digestibility was measured using an in vitro Englyst test [11]. The procedure involved placing a test tube containing feed samples, glass balls, digestive enzymes, and incubation buffers in an oscillating water bath (37 °C; two tubes per sample). After incubating the samples for 12 time periods (0 min, 5 min, 10 min, 15 min, 20 min, 30 min, 45 min, 60 min, 90 min, 120 min, 180 min, and 240 min), they were aliquoted, and the glucose quantity was determined using a colorimetric method. The above operations were carried out according to the experiment conducted by Yang et al. [12].

According to the kit instructions, glucose standards of different concentration gradients were prepared, and the standard curve was plotted, y = 0.5345x + 0.0016 (R2 = 0.999), substituting x (OD value) to obtain y (glucose concentration).

Starch hydrolysis rate (%) = (G × 0.9)/m × 100

G indicates the amount of glucose produced after hydrolysis; 0.9 is the conversion coefficient of starch-hydrolyzed glucose; and m is the total starch content (mg).

### 2.2. In Vivo Study

#### 2.2.1. Experimental Design

We obtained 240 healthy 35-day-old male Jiangnan White goslings from Changzhou Four Seasons Poultry Industry Co., Ltd. (Jintan, China). The birds were randomly allocated to four dietary treatment groups (n = 6 replicates/group; 10 birds/replicate). The geese received iso-energetic diets (Table 1) with different starch sources: GNR (glutinous rice; high amylopectin, negligible amylose), CRN (corn), INR (indica rice; high amylose), and CRN + AM (amylose-supplemented). The 33-day trial concluded when the goslings reached 68 days of age. Throughout the study, ad libitum access to feed and water was provided using plastic-lined pens with elevated feeders (70 cm height; 2 cm^2^ mesh openings) and semi-open cylindrical water tanks. The environmental conditions were maintained at 26.0 ± 3.0 °C, 65.5% ± 5.0% relative humidity, a 16 h light/dark cycle, and a stocking density of 0.5 m^2^/bird.

#### 2.2.2. Continuous Blood Glucose Monitoring

On the 59th day of the experiment, a single goose exhibiting a body weight closest to the average was selected from each pen and transferred to an individual stainless-steel metabolic cage for continuous blood glucose monitoring. Blood glucose concentrations were measured following a fasting period and subsequently at 15, 30, 60, 120, 180, 240, and 300 min post-feeding. The rate of blood glucose increase (mg/dL/min) was calculated as the quotient of the change in the blood glucose concentration divided by the time elapsed between any two designated measurement points. All blood glucose measurements were performed using a commercial glucometer (Simcere Zaikang Jiangsu Pharmaceutical Co., Ltd., Nanjing, China).

#### 2.2.3. Nutrient Utilization Rate

At 63 days of age, a single gosling per replicate was selected, which had an individual body weight closest to the group’s mean body weight. The selected geese were individually housed in metabolic cages (75 cm × 65 cm × 35 cm) featuring wire flooring. Each cage was equipped with an individual feeder and a self-service water system. A 3-day pretest period preceded the main trial, during which the birds had ad libitum access to feed and water. This was immediately followed by the 3-day main experimental period. Prior to initiating the main experiment, the geese underwent 24 h of fasting. They were then provided ad libitum access to their respective experimental diets. Feces were quantitatively collected over the subsequent 72 h using the total collection method [13]. The collected excreta were deposited into plastic trays; to preserve their nitrogen content, 10 mL of 10% hydrochloric acid was added per 100 g of fresh feces. Both the fecal samples and the corresponding experimental diet samples were dried in a forced-air oven at 65 °C. After drying, the samples were equilibrated to ambient moisture for 24 h under natural conditions, ground to pass completely through a 40-mesh sieve, and stored at −20 °C pending further analysis.

#### 2.2.4. Determination Method

Starch quantification was performed using a commercial assay kit (Nanjing Jiancheng Bioengineering Institute, Nanjing, China), following the manufacturer’s protocol. Crude protein was determined via the Kjeldahl method using a FoodALYT D1000 Nitrogen Analyzer (FoodALYT, Bremen, Germany). Crude fat content was assessed via solvent extraction using a FoodALYT RD40 Fat Analyzer (FoodALYT, Germany). Amino acid analysis involved ion-exchange chromatography with post-column ninhydrin derivatization using a Hitachi L-8900 automatic amino acid analyzer (Tokyo, Japan), quantifying seventeen amino acids: aspartic acid (Asp), threonine (Thr), serine (Ser), glutamic acid (Glu), glycine (Gly), alanine (Ala), cysteine (Cys), valine (Val), methionine (Met), isoleucine (Ile), leucine (Leu), tyrosine (Tyr), phenylalanine (Phe), lysine (Lys), histidine (His), arginine (Arg), and proline (Pro).

The apparent utilization calculation method was as follows:

Utilization rate of a certain nutrient (%) = (Feed intake × Nutrient diet − Excreta output × Nutrient excreta)/Feed intake × Nutrient diet) × 100%

### 2.3. Statistical Analysis

The data were initially compiled using Microsoft Excel 2020. Further statistical analyses were executed using SPSS Statistics software (Version 20.0, SPSS Inc., Chicago, IL, USA). Blood glucose measurements underwent analysis via a repeated-measures analysis of variance (ANOVA) based on a randomized block design, utilizing a 4 (dietary treatments) × 7 (time periods) factorial model. This model assessed the influence of the primary starch sources (four dietary groups), the seven sampling times, and their potential interaction. When the ANOVA results indicated statistically significant effects (*p <* 0.05), Tukey’s honestly significant difference (HSD) test was applied for post hoc pairwise comparisons. All outcomes are expressed as the mean ± standard error of the mean (SEM).

## 3. Results

### 3.1. Starch Particle Size and Morphology

**Figure 1:**
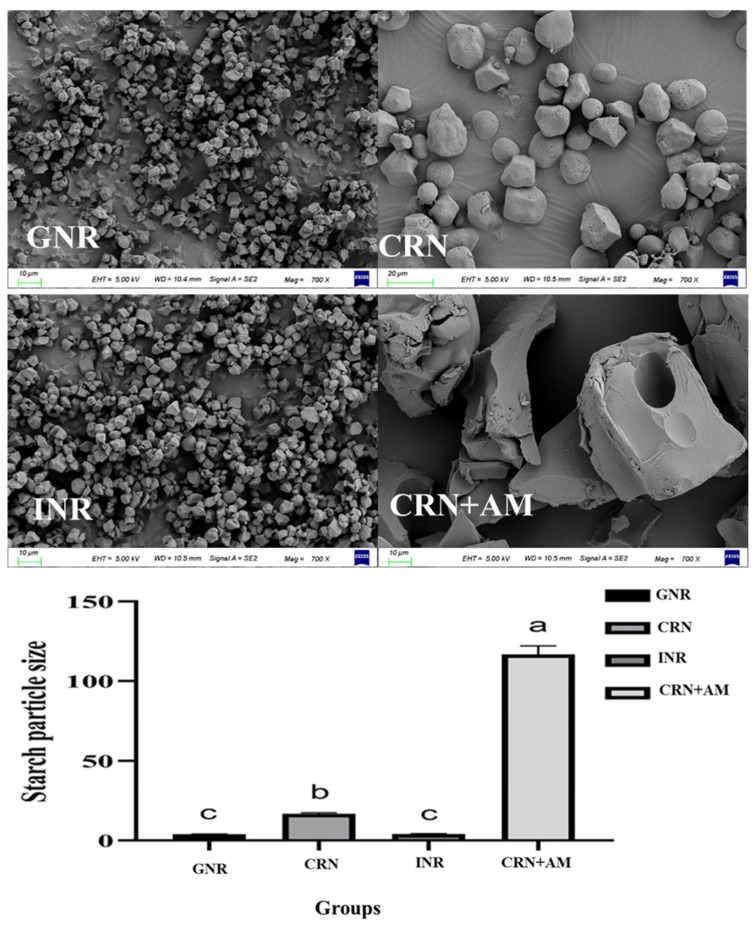
SEM showing starch particle sizes from different dietary source ^1,2^. ^1^ Different lowercase letters indicate significant differences among groups (*p* < 0.05). ^2^ GNR (glutinous rice; high amylopectin, negligible amylose), CRN (corn), INR (indica rice; high amylose), and CRN + AM (amylose-supplemented). Values represent the mean diameter in micrometers (μm).

**Figure 2:**
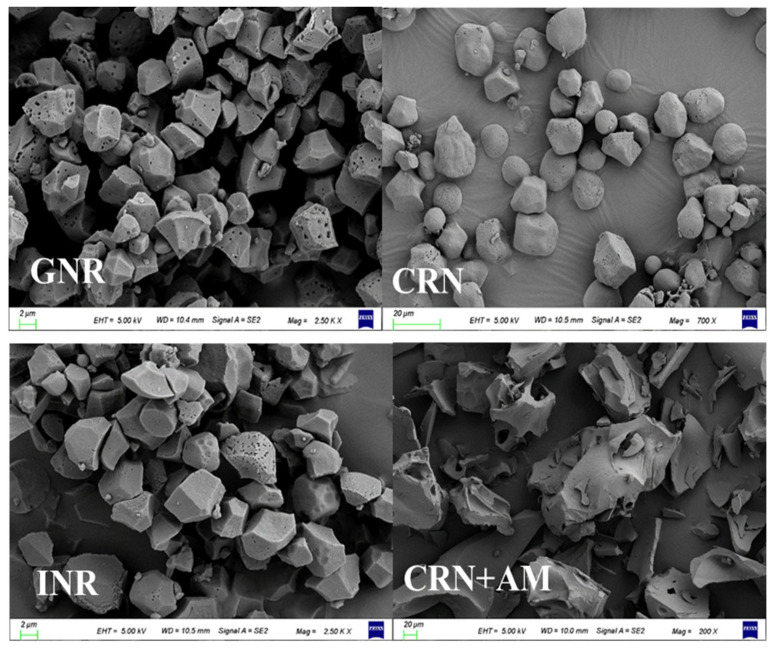
SEM of morphological structure of starch granules from different dietary source ^1^. ^1^ GNR (glutinous rice; high amylopectin, negligible amylose), CRN (corn), INR (indica rice; high amylose), and CRN + AM (amylose-supplemented).

The results showed that the starch particle sizes differed among the dietary raw materials. The sizes of the starch grains from glutinous rice and indica rice were similar but smaller than the maize and high-amylose starch (*p* < 0.05). The maize starch particles were larger than those of the glutinous rice and indica rice but smaller than the high-amylose starch. The high-amylose starch particles were larger than the glutinous rice, indica rice, and maize starch particles, as shown in Figure 1. The grain morphology varied significantly as well. The glutinous rice starch grain was a porous polyhedron with an angular surface, the corn starch was an ellipsoid with a smooth surface, the indica rice starch was a polyhedron with a smooth and compact surface, and the high-amylose starch was an irregular polyhedron with a smooth surface (Figure 2).

### 3.2. In Vitro Starch Digestion Rate

**Figure 3:**
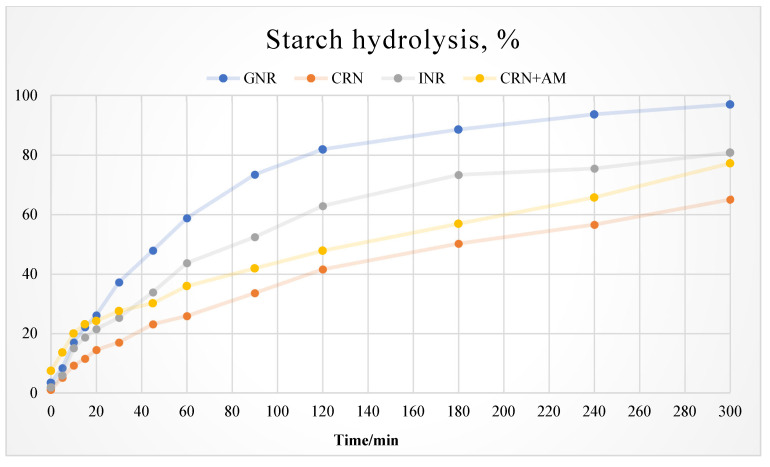
Starch digestion patterns of diets with different starch sources ^1^. ^1^ GNR (glutinous rice; high amylopectin, negligible amylose), CRN (corn), INR (indica rice; high amylose), and CRN + AM (amylose-supplemented).

The experimental results showed that the rates of digestion of the different starch sources were significantly different. The digestibility of the starch in the glutinous feed grain was very high, with a significant variation rate. Digestibility was relatively stable for the indica- and corn-based diets, although the rate of digestion of the indica rice diet was higher than that of the corn and high amylose-based diets, as shown in Figure 3.

### 3.3. In Vivo Blood Glucose Monitoring

The time after feeding significantly affected the speed of blood glucose elevation (*p* < 0.05). The blood glucose concentration increased from fasting to 30 min after feeding and decreased from 30 min to 300 min after feeding. The most pronounced surge in blood glucose concentration occurred 15 to 30 min after feeding (Table 2).

The dietary starch source effectively influenced the postprandial blood glucose response rate across the measured time intervals. Specifically, the glutinous rice group exhibited a comparatively higher rate of blood glucose concentration than the other groups during the 15- to 30-min interval postprandially (*p* < 0.05). Subsequently, between 30 and 60 min post-feeding, blood glucose concentrations began to decline in the glutinous rice, corn, and indica rice groups, while the rate of increase decelerated in the high-amylose rice group (Table 3).

### 3.4. Growth Performance

Compared to the high amylose starch group, the geese that consumed the corn or indica rice diets achieved a significantly greater body weight (BW) by day 63 (*p* < 0.05). However, no significant differences in the feed-to-gain ratio (F/G) were observed across dietary treatments, as reported previously [14].

### 3.5. Nutrient Digestibility

The glutinous rice group exhibited superior total starch digestibility compared to the groups consuming other starch sources (*p <* 0.05) and significantly higher crude protein utilization than the corn and high-amylose rice groups. Crude fat utilization remained unaffected by the starch source (*p >* 0.05) (Table 4).

### 3.6. Amino Acid Availability

The effects of diets incorporating different starch sources on amino acid availability in goslings are presented in Table 5. Statistical analysis revealed significant differences (*p <* 0.05) in the availability of the following amino acids: aspartic acid (Asp), threonine (Thr), serine (Ser), glutamic acid (Glu), glycine (Gly), alanine (Ala), valine (Val), methionine (Met), isoleucine (Ile), leucine (Leu), tyrosine (Tyr), phenylalanine (Phe), proline (Pro), and lysine (Lys). Specifically, goslings fed the glutinous rice-based diet exhibited significantly higher availability of Asp, Ser, Glu, Gly, and Phe compared to those that received corn-based or high-amylose rice-based diets. Furthermore, Thr and Ala availability was significantly greater in the glutinous rice group than the indica rice- or high-amylose rice-based groups. Methionine availability was higher in the goslings that consumed the corn and glutinous rice-based diets relative to those that consumed the indica rice- or high-amylose rice-based diets. Lysine availability was elevated in the indica and glutinous rice groups compared to the corn and high-amylose rice groups. Finally, proline utilization was significantly higher in the corn-based diet group than in all other groups.

## 4. Discussion

There are significant variations in the starch contained in different grains. The results of this experiment showed significant differences in the particle size, shape, and structure of starch grains from different cereals (Figure 1 and Figure 2). The size of the starch grains and their structure are important factors affecting starch digestion. Studies have shown that the larger the starch granule, the smaller its relative surface area, which reduces the effective contact area between starch and amylase, thereby decreasing enzymatic decomposition efficiency [14,15]. These structural variations are fundamentally linked to starch composition: glutinous rice (>99% amylopectin) exhibits branched-chain clustering that creates porous architectures, while indica rice (15–25% amylose) forms dense crystalline regions through linear polymer packing [16]. The hydrolysis of starch affects glucose uptake in the intestine, driving blood glucose dynamics [17,18]; Figure 1 and Figure 2 and Table 3 verify this conclusion. The small starch particles of glutinous and indica rice correlate with the rapid glycemic responses observed following the consumption of these types of rice. Critically, despite their similar granule sizes, the higher glycemic index of glutinous rice stems from its amylopectin-dominated composition, which enables porous surface structures [19], facilitating amylase penetration. Conversely, the amylose-rich composition of indica rice promotes tight granule organization with minimal surface stomata [20], impeding enzymatic hydrolysis.

In the in vitro digestion tests (Figure 3), we observed that the starch hydrolysis rate for the glutinous rice diet was unstable, showing a rapid increase followed by a rapid decrease. The digestion rates of the indica, corn, and high amylose rice diets were more stable, with the indica diet exhibiting high, consistent digestibility. This aligns with the in vivo blood glucose results (Table 3). However, starch hydrolysis kinetics diverged between the in vitro and in vivo systems: in vivo, blood glucose spiked 0–30 min post-feeding, while in vitro hydrolysis remained low during this period. Hasjim et al. [21] attributed this difference to in vivo complexities (mastication, intestinal motility, and enzyme regulation). We further propose that the amylose–lipid complexes formed during in vivo digestion [20,22] delay glucose absorption—a process that is not replicated in vitro—while salivary amylase rapidly erodes amylopectin surfaces in glutinous rice, accelerating early-stage glycemic responses [23].

Blood glucose is a critical postprandial metabolic indicator [21]. The results in Table 2 show time-dependent blood glucose change rates in the goslings: a rapid increase (0–30 min) followed by a decline after 30 min. This suggests that glucose-lowering mechanisms (e.g., insulin secretion [23]) surpass the glucose-raising capacity post-30 min, while intestinal glucose generation from starch hydrolysis dominates earlier phases [21,22].

Starch-derived glucose and amino acids serve as energy sources in the primary digestive tract [24]. A timely glucose supply reduces amino acid oxidation, sparing protein. Our results (Table 4) showed that the glutinous rice diet resulted in higher total starch and crude protein utilization. This aligns with the amylopectin-driven properties of glutinous rice: its small granule size and porous structure enhance digestibility [25], while the rapid front-loaded release of glucose in the proximal intestine spares amino acids from oxidation. Paradoxically, this creates distal glucose deficits [26,27], necessitating amino acid catabolism for energy, explaining why goslings fed the glutinous rice diet exhibited lower cysteine, proline, histidine, and arginine (Table 5) utilization. These exceptions may arise for [28] the following reasons: cysteine is prioritized for protein synthesis over oxidation due to disulfide bond formation [29]; proline‘s unique mitochondrial catabolism less tightly coupled to glucose availability [30]; and histidine/arginine are reserved for signaling molecule synthesis (e.g., histamine, nitric oxide) [31,32,33].

Thus, while most amino acids benefited from rapid glucose flux, these functionally distinct AAs were conserved for non-energy roles. The amylose–amylopectin ratio emerges as a master regulator of nutrient partitioning: high-amylopectin starches (glutinous rice) maximize proximal glucose flux but risk distal amino acid oxidation, and high-amylose starches (indica/corn) enable sustained glucose release, balancing AA utilization but limiting peak protein synthesis potential.

## 5. Conclusions

This study showed that the particle size and structure of starch from different sources (glutinous rice, corn, indica rice, and high-amylose rice) vary. The smaller the starch particle size and the looser the structure, the faster the hydrolysis process. The glutinous rice diet contains more rapidly digestible starch, which is quickly digested in the proximal intestine, and the inadequate supply of glucose in the distal intestine enhances the oxidative energy supply from dietary amino acids in that region, thereby improving the apparent digestibility of both starch and crude protein. Therefore, corn and indica rice are more suitable starch sources for providing energy to geese. However, further studying the differences and complementarity of starch and protein in intestinal digestion and rational utilization can improve gosling diets.

## Figures and Tables

**Table 1:** Energy and nutrient composition (dry basis).

Items ^1^	Groups			
	GNR	CRN	INR	CRN + AM
Ingredients, %				
Corn	-	57.60	-	49.40
Indica rice	-	-	53.75	-
Glutinous rice	51.00	-	-	-
Amylose	-	-	-	6.00
Soybean meal	23.20	24.20	23.45	25.00
Rice husk	9.60	9.50	10.10	9.30
Bran	12.45	4.90	9.05	6.40
Stone powder	1.10	1.10	0.90	1.20
Calcium hydrogen	1.20	1.10	1.30	1.10
Salt	0.30	0.30	0.30	0.30
Lysine	-	0.10	-	0.10
DL-methionine	0.15	0.20	0.15	0.20
Premix ^2^	1.00	1.00	1.00	1.00
Total	100.00	100.00	100.00	100.00
Nutritional level ^3^				
Energy (ME, MJ/kg)	10.86	10.82	10.80	10.80
Crude protein (%)	15.93	16.18	16.01	16.14
Crude fiber (%)	6.86	6.87	6.83	6.80
Lysine (%)	0.91	0.91	0.90	0.92
Methionine (%)	0.41	0.43	0.42	0.43
Total phosphorus (%)	0.61	0.59	0.60	0.61
Calcium (%)	0.85	0.82	0.83	0.86
Total starch (%)	37.92	38.84	39.77	38.16
Amylose/Amylopectin ratio	0.021	0.340	0.456	0.568

^1^ GNR (glutinous rice; high amylopectin, negligible amylose), CRN (corn), INR (indica rice; high amylose), and CRN + AM (amylose-supplemented). ^2^ The vitamin–mineral premix, supplied at 1 kg per diet tonne, contained Vitamin A (9,000,000 IU), Vitamin D (300,000 IU), Vitamin E (1800 IU), Vitamin K (150 mg), Vitamin B_1_ (90 mg), Vitamin B_2_ (800 mg), Vitamin B_6_ (320 mg), Vitamin B_12_ (1.2 mg), nicotinic acid (4.5 g), pantothenic acid (1100 mg), folic acid (65 mg), biotin (5 mg), Fe (6 g as ferrous sulfate), Cu (1 g as copper sulfate), Mn (9.5 g as manganese sulfate), Zn (9 g as zinc sulfate), I (50 mg as potassium iodide), and Se (30 mg as sodium selenite). ^3^ The values reported for crude protein, calcium, total phosphorus, and total starch were determined analytically; all other compositional values are calculated estimates based on the formulated diet composition.

**Table 2:** Effects of diets with different starch sources and time after feeding on rate of blood glucose increase in goslings ^1^.

Item	Blood Glucose Increase Rate, mmol/L/min
Time, min	
s~15 ^2^	0.123 ^a^
15~30	0.052 ^b^
30~60	−0.009 ^c^
60~120	−0.023 ^c^
120~180	−0.023 ^c^
180~240	−0.007 ^c^
240~300	−0.011 ^c^
SEM	0.005
Starch source	
Glutinous rice diet	0.020
Corn diet	0.017
Indica rice diet	0.015
High-amylose diet	0.017
SEM	0.005
*p*-value	
Time	<0.001
Linear	<0.001
Quadratic	<0.001
Starch source	0.988
Time × Starch source	<0.001

^a,b,c^ Within each row, statistically distinct values (*p* < 0.05) are marked with differing lowercase superscript letters, while statistically similar values (*p* > 0.05) share identical superscript letters. ^1^ All data points represent mean values from six replicate measurements. The dash symbol (−) denotes a measured decrease. ^2^ “S~15” means from start of feeding to 15 min after feeding.

**Table 3:** Interactive effects of starch source and time after feeding on rate of blood glucose in goslings ^1,2,3^.

Time, min	Groups, mmol/L/min				SEM	*p*-Value
	GNR	CRN	INR	CRN + AM		
s~15 ^4^	0.194 ^a^	0.089 ^b^	0.112 ^b^	0.098 ^b^	0.011	<0.001
15~30	0.072 ^a^	0.050 ^b^	0.044 ^b^	0.042 ^b^	0.004	0.013
30~60	−0.038 ^c^	0.000 ^ab^	−0.010 ^b^	0.012 ^a^	0.005	<0.001
60~120	−0.034	−0.018	−0.016	−0.022	0.006	0.759
120~180	−0.012	0.010	−0.008	−0.016	0.004	0.256
180~240	−0.006	−0.014	−0.010	0.002	0.005	0.714
240~300	−0.012	−0.020	−0.006	−0.006	0.005	0.691

^1^ GNR (glutinous rice; high amylopectin, negligible amylose), CRN (corn), INR (indica rice; high amylose), and CRN + AM (amylose-supplemented). ^2^ Within each row, statistically distinct values (*p* < 0.05) are marked with differing lowercase superscript letters, while statistically similar values (*p* > 0.05) share identical superscript letters. ^3^ All data points represent mean values from six replicate measurements. The dash symbol (−) denotes a measured decrease. ^4^ “S~15” means from the start of feeding to 15 min after feeding.

**Table 4:** Apparent nutrient digestibility in goslings fed diets with varying starch sources ^1,2,3^.

Item, %	Groups				SEM	*p*-Value
	GNR	CRN	INR	CRN + AM		
Total starch	92.24 ^a^	89.94 ^b^	90.01 ^b^	89.30 ^b^	0.324	0.002
Crude protein	87.64 ^a^	81.32 ^b^	85.30 ^ab^	81.44 ^b^	0.882	0.015
Crude fat	81.76	83.43	80.73	83.23	0.874	0.776

^1^ GNR (glutinous rice; high amylopectin, negligible amylose), CRN (corn), INR (indica rice; high amylose), and CRN + AM (amylose-supplemented). ^2^ Within each row, statistically distinct values (*p* < 0.05) are marked with differing lowercase superscript letters, while statistically similar values (*p* > 0.05) share identical superscript letters. ^3^ All data points represent mean values from six replicate measurements.

**Table 5:** Gosling amino acid digestibility responses to various starch diets ^1,2,3^.

Item, %	Groups				SEM	*p*-Value
	GNR	CRN	INR	CRN + AM		
Asp	91.79 ^a^	87.12 ^b^	89.66 ^ab^	87.69 ^b^	0.565	0.004
Thr	88.78 ^a^	86.12 ^ab^	85.77 ^b^	82.63 ^c^	0.647	0.002
Ser	91.82 ^a^	87.54 ^c^	90.10 ^ab^	88.12 ^bc^	0.513	0.003
Glu	93.66 ^a^	91.40 ^b^	92.27 ^ab^	90.99 ^b^	0.354	0.024
Gly	86.35 ^a^	80.08 ^bc^	83.21 ^ab^	75.88 ^c^	1.137	0.001
Ala	86.42 ^a^	84.93 ^ab^	81.94 ^b^	81.79 ^b^	0.649	0.011
Cys	75.19	81.38	67.99	77.44	2.117	0.146
Val	88.01 ^a^	83.73 ^b^	83.96 ^b^	81.29 ^b^	0.742	0.004
Met	95.50 ^a^	94.75 ^a^	84.34 ^b^	84.54 ^b^	1.263	<0.001
Ile	91.61 ^a^	85.79 ^b^	87.28 ^b^	85.01 ^b^	0.747	0.001
Leu	90.97 ^a^	89.36 ^ab^	89.24 ^ab^	87.11 ^b^	0.472	0.022
Tyr	91.45 ^a^	89.34 ^a^	89.01 ^a^	80.22 ^b^	1.077	<0.001
Phe	92.30 ^a^	86.62 ^c^	90.13 ^ab^	88.22 ^bc^	0.616	0.001
Lys	90.76 ^a^	86.45 ^b^	89.81 ^a^	86.91 ^b^	0.581	0.006
His	96.73	95.76	94.88	93.67	0.501	0.163
Arg	95.38	92.46	93.25	95.20	0.520	0.113
Pro	86.17 ^b^	90.53 ^a^	81.24 ^c^	86.03 ^b^	0.970	0.002

^1^ GNR (glutinous rice; high amylopectin, negligible amylose), CRN (corn), INR (indica rice; high amylose), and CRN + AM (amylose-supplemented). ^2^ Within each row, statistically distinct values (*p* < 0.05) are marked with differing lowercase superscript letters, while statistically similar values (*p* > 0.05) share identical superscript letters. ^3^ All data points represent mean values from six replicate measurements. Asp: aspartic; Thr: threonine; Ser: serine; Glu: glutamic; Gly: glycine; Ala: alanine; Cys: cysteine; Val: valine; Met: methionine; Ile: isoleucine; Leu: leucine; Tyr: tyrosine; Phe: phenylalanine; Lys: lysine; His: histidine; Arg: arginine; Pro: proline.

## Data Availability

The original contributions presented in this study are included in the article. Further inquiries can be directed to the corresponding author.
